# B cell lines fail to support efficient rhesus enteric calicivirus and human norovirus replication

**DOI:** 10.1128/jvi.00143-25

**Published:** 2025-04-22

**Authors:** Tibor Farkas

**Affiliations:** 1 Department of Veterinary Pathobiology, College of Veterinary Medicine & Biomedical Sciences, Texas A&M University 199062 , College Station, Texas, USA; University of Michigan Medical School, Ann Arbor, Michigan, USA

**Keywords:** B cell, rhesus enteric calicivirus, histo-blood group antigen, Coxsackie and adenovirus receptor

## Abstract

Analyses of intestinal biopsies of infected individuals and/or nonhuman primates (NHP) suggested the possible immune cell tropism of human noroviruses (HuNoV) and rhesus enteric caliciviruses (ReCV). Subsequently, the first HuNoV cell culture system using human B cell lines was reported. However, reproducibility issues raised questions about the validity and suitability of B cell cultures for HuNoV research. Histo-blood group antigens (HBGA) are known HuNoV susceptibility factors, but the full range of HuNoV susceptibility determinants remains unknown. In contrast, strain-specific ReCV susceptibility determinants have been recently characterized. Here, we evaluated NHP B cell lines and the human BJAB cell line for susceptibility to ReCV-FT285 infection, which is controlled by the Coxsackie and adenovirus receptor (CAR) and the type A or B HBGA. NHP B cell lines lacked CAR and HBGA expression and resisted infection. Inconsistent, low-level virus replication was detectable in BJAB cells, and expression of CAR and HBGAs was evident by Western blots. However, <1% of live, but >80% of fixed and permeabilized BJAB cells were CAR+, suggesting that CAR is mostly internalized. Co-transfection of BJAB cells with hCAR and A enzyme expression vectors led to substantial surface CAR and type A HBGA expression but not to an increase in ReCV titers. dsRNA staining revealed initial ReCV and HuNoV infection in a few cells that most likely became abortive. Based on both the similarities between ReCV and HuNoV replication profiles and the results obtained in the present study, considering BJAB cells an efficient culture system for HuNoV research is not justified.

**IMPORTANCE:**

Recently, two human norovirus (HuNoV) cell culture systems have been developed—the B cell culture system and the enteroid culture system. While the enteroid cell culture system became widely used in HuNoV research, mainly due to reproducibility issues, the B cell culture system did not. Here, we used HuNoV and rhesus enteric caliciviruses (ReCV) to evaluate enteric calicivirus B cell infections, in correlation to cell surface molecular determinants that control the susceptibility to infection. These are fully characterized for ReCVs, but not for HuNoVs. We found that only few BJAB cells express the cell surface molecules necessary for ReCV infection and support low-level, initial ReCV and HuNoV infection, but virus replication is most likely abortive, with minimal progeny virus release. Our findings and the poor reproducibility indicate that the B cell culture system in its current form is unsuitable for ReCV or HuNoV research and does not represent an efficient valid cell culture system.

## INTRODUCTION

Human norovirus (HuNoV) infection is one of the leading causes of acute gastroenteritis and severe childhood diarrhea globally. It also accounts for most food- and water-borne gastroenteritis outbreaks in developed countries (1, 2). The cellular tropism of HuNoVs has long been a subject of debate. The concept of HuNoV immune cell tropism emerged following the discovery of murine norovirus (MNV) as the first cell culture-propagable norovirus with a tropism for dendritic cells and macrophages (3). Subsequent studies, including animal challenges and analysis of patient biopsy samples indicated the presence of HuNoV antigens in immune cells, such as dendritic cells, macrophages, and CD19+/CD20 +B cells, of the intestinal lamina propria (4–6). The definitive indicator of HuNoV immune cell tropism was provided by Jones et al. in 2014, who documented productive HuNoV infection in various B cell lines and reported the establishment of the first HuNoV cell culture system (7, 8). However, due to low virus yields and inconsistent reproducibility, the use of this system did not gain acceptance in the field, and the suitability of the BJAB cell culture system for HuNoV research is questioned.

Several other HuNoV culture systems have also been reported (9–13), from which the human intestinal stem cell-derived enteroid culture system, reported in 2016 by Ettayebi et al*.*, became the most widely used worldwide (10). However, even the enteroid culture system has its limitations, including the requirement for stool-derived virus, the relatively low yield compared to other cell culture systems, the time-consuming procedures, and the high cost that limits its use in many laboratories. Thus, the development of a simple and efficient cell culture system allowing the continuous propagation of HuNoVs and the generation of high-yield virus stocks still remains a challenging goal (14). Unfortunately, the limited insight into the cellular factors influencing HuNoV infection susceptibility hampers our capacity to further assess and refine the HuNoV cell culture systems.

In 2012, we reported the presence of Tulane virus antigen-positive B cells in the duodenal lamina propria of experimentally infected macaques and a modest viral genome copy number increase that was likely associated with CD20 +cells in rhesus PBMC cultures, indicating a possible B cell tropism of recoviruses (ReCV) (15). More recently, we identified the Coxsackie and adenovirus receptor (CAR) as the ReCV entry receptor and characterized strain-specific requirements for cell surface expression of CAR and the type A or B HBGAs or sialic acid as the minimum requirements to promote susceptibility to ReCV infections (16, 17). To gain valuable insights into enteric calicivirus B cell tropism, here, we assessed ReCV infection in three nonhuman primate (NHP) B cell lines and the human BJAB cell line in correlation with the cell surface expression of strain-specific susceptibility markers.

## RESULTS

### NHP B cell lines do not support ReCV replication and lack detectable CAR or HBGA expression

**Fig 1:**
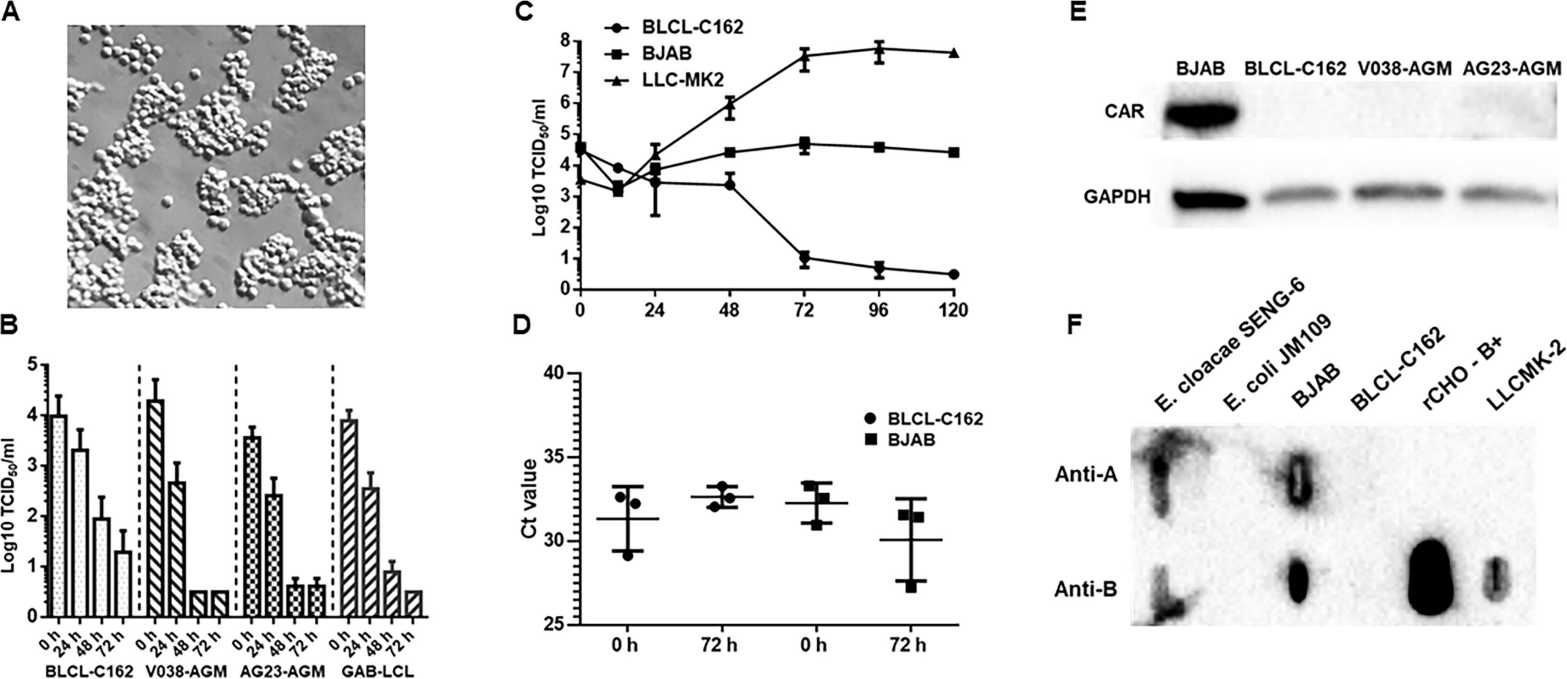
BJAB cells but not the NHP B cell lines express CAR and support very low levels of ReCV replication. (**A**) Healthy B cell culture (BJAB cells are shown) with round cells displaying a sharp, shiny periphery and forming small clumps of 25–50 cells. (**B**) Titration of ReCV-FT285-infected (1 MOI) NHP B cell cultures in LLC-MK2 monolayers. No virus replication is indicated. (**C**) Titration of ReCV- FT285-infected (1 MOI) BLCL-C162 (rhesus) and BJAB (human) cell cultures in LLC-MK2 monolayers. A low level of virus replication is indicated in the BJAB but not in the BLCL-C162 cell line by the eclipse at 12 hpi and increasing titers. Note the different kinetics between the two cell lines. (**D**) qRT-PCR analysis of ReCV-infected BLCL-C162 and BJAB cell cultures. Ct values are shown. A low level of virus replication is indicated in the BJAB but not in the BLCL-C162 cell line by the decrease in Ct values at 72 hpi. (**E**) Western blot analysis for CAR expression. Only the BJAB cell lines but not the NHP B cell lines displayed detectable CAR expression. GAPDH was used as a loading control. (**F**) Anti-A and anti-B monoclonal antibodies detected the presence of both type A and type B HBGAs in slot blots of BJAB cell lysates and in the positive control rCHO-B +cells, but not in the NHP cell lines (BLCL-C162 is shown). One representative of three independent experiments using the BJAB-LSU cells is shown. BJAB-LSU and BJAB-CDC yielded similar results.

None of the NHP B cell lines used in this study (see Materials and Methods) supported ReCV replication, as evidenced by the lack of increase in virus titers between 0 and 72 hours post-infection. Indeed, a gradual decrease in virus titers was observed in these cultures (Fig. 1B). Recently, we demonstrated that the cell surface expression of CAR and either the type A or B HBGA is an absolute requirement for ReCV-FT285 infection (17). Western blot analysis revealed the lack of CAR or HBGA expressions in all NHP B cell lines included in our study (Fig. 1E and F).

### BJAB cell line supports initial ReCV infection

In the BJAB cultures, a slight decrease (21-fold) in infectious virus titers was evident at 12 hours post-infection (4.31E + 03 TCID^50^/mL), followed by a gradual increase that plateaued at 72 hours (9.96 ± 5.14E + 04 TCID^50^/mL) but did not reach a higher titer than the initial titer at 0 hour (7.8E ± 2.09E + 04 TCID^50^/mL) (Fig. 1C). In contrast, titers in the BCL-162 cultures gradually declined to a level below the detection limit of the assay (<10 TCID^50^/mL) by 120 hours post-infection. To corroborate this observation, in separate experiments, we evaluated viral genome copy numbers at 0 hour and 72 hours post-infection in ReCV-FT285-infected BCL-C162 and BJAB cultures. Changes in Ct values indicated a similar outcome as the TCID_50_ assay. While in the BCL-C162 cultures Ct values increased at 72 hours post-infection (mean: 1.3 ± 1.65, indicating a lower viral load), in the BJAB cultures, Ct values at 72 hours post-infection were slightly lower than at 0 hour (mean: −2.2 ± 1.57), indicating a higher viral load (Fig. 1D). None of these changes were statistically significant; however, the different kinetics of virus titers and genome copy numbers between the NHP B cell lines and the BJAB cell line and the clearly detectable eclipse at 12 hours post-infection indicated modest (<10 fold increase) infection and possibly virus replication in the BJAB cultures. Western blot and slot blot analysis revealed the presence of CAR and type A and B HBGAs in the BJAB cell lysates but not in the BLCL-C162 rhesus B cell line or in the other NHP B cell lines included in this study (Fig. 1E and F).

### CAR remains internalized in BJAB cells

**Fig 2:**
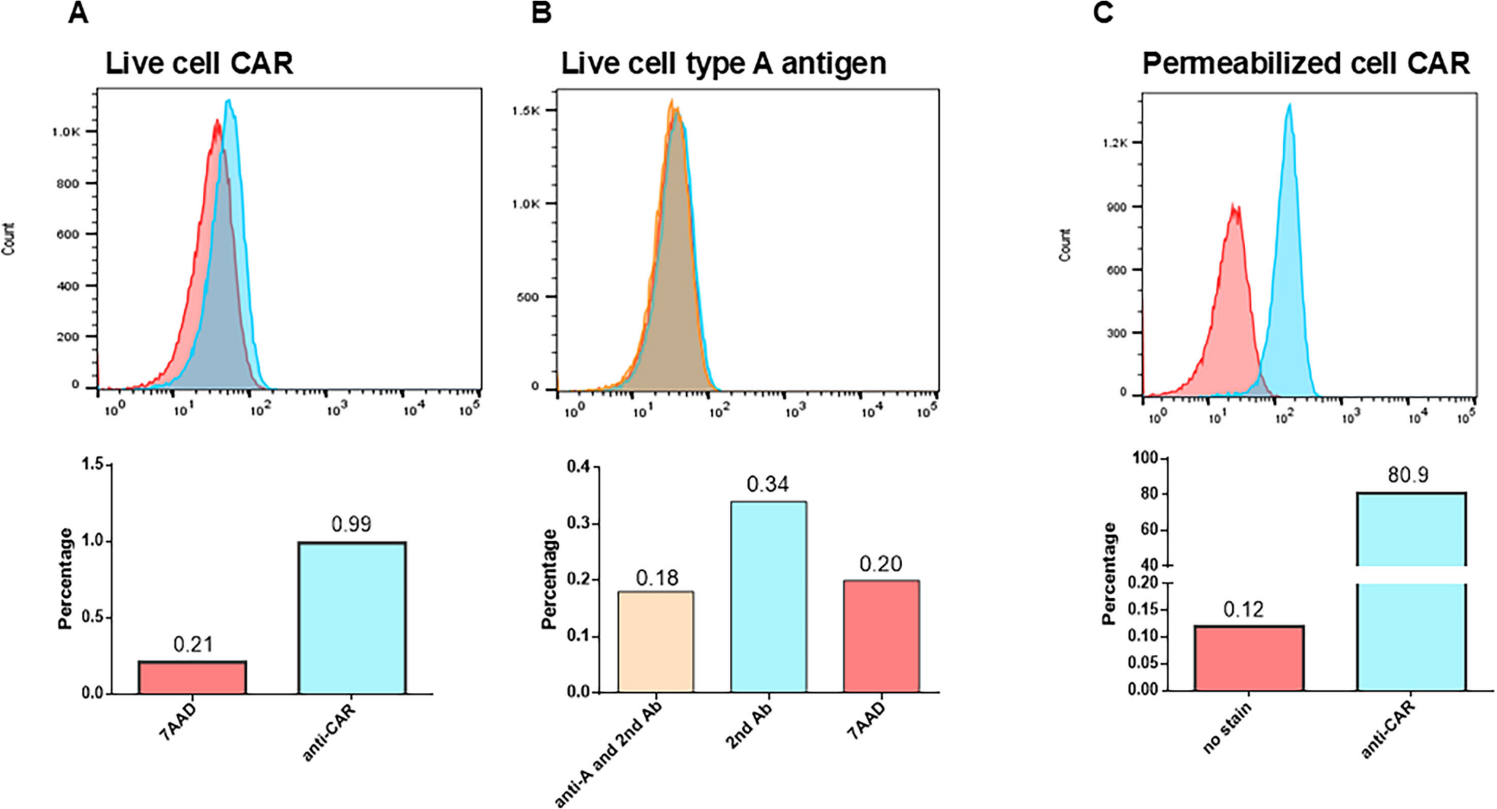
Flow cytometry analysis of BJAB cells indicated that CAR remains internalized. (**A**) Live cell staining revealed that <1% of cells display CAR on the cell surface. (**B**) No cell surface expression of HBGAs could be detected (data for the type B antigen are not shown). (**C**) Analysis of fixed and permeabilized BJAB cells revealed CAR expression in >80% of the cells. One representative of three independent experiments with BJAB-LSU is shown. BJAB-CDC yielded similar results.

Flow cytometry analysis of live cells revealed that <1% of the BJAB cells display CAR or the type A/B HBGAs on the cell surface (Fig. 2A and B). On the other hand, >80% of fixed and permeabilized BJAB cells showed CAR expression (Fig. 2C), indicating that while CAR is expressed (Fig. 1E), in most of the cells, it remains internalized and inaccessible for virus entry. Similar results were obtained for both BJAB cell line clones (LSU and CDC).

**Fig 3:**
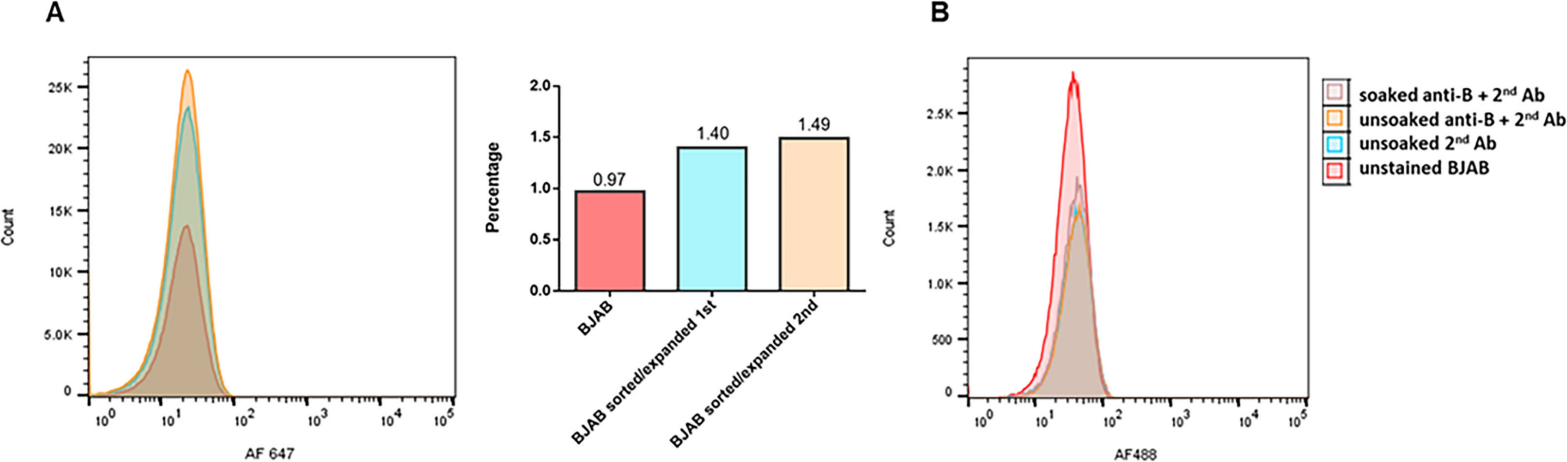
The cell surface CAR- or HBGA-positive cell population did not increase after two rounds of sorting and expansion or incubating cells in human AB serum, respectively. (**A**) Live cell staining of the original or sorted and expanded CAR-positive cell populations with anti-CAR-FITC antibody. No enrichment of CAR-positive cells was achieved. (**B**) Live cell staining of the original (unsoaked) and human AB serum-soaked BJAB cell populations with anti-B mouse Mab and AF488-labeled secondary antibody. Soaking BJAB cells in human AB serum did not increase the number of HBGA-positive cells. One representative of three independent experiments with the BJAB-LSU cell clone is shown.

Our attempts to enrich the cell surface CAR-positive cell population by repeated rounds of cell sorting and expansion were unsuccessful. The number of CAR +live cells always readjusted to ~1% in the cell population (Fig. 3A). We are unable to provide a valid explanation for this phenomenon, but it seems the ratio of cell surface CAR +cells is controlled.

The discrepancy between slot blot detection (Fig. 1F) of HBGA expression and live cell staining (Fig. 2B) was not investigated since CAR is a general requirement for all ReCV strains, while HBGAs are not (13). We attempted to enrich the membrane-associated HBGAs by soaking and culturing BJAB cells in human AB serum but did not observe an increase in live cell staining (Fig. 3B).

### Recombinant BJAB cells with enriched cell surface expression of CAR and type A HBGAs failed to support efficient ReCV infections

**Fig 4:**
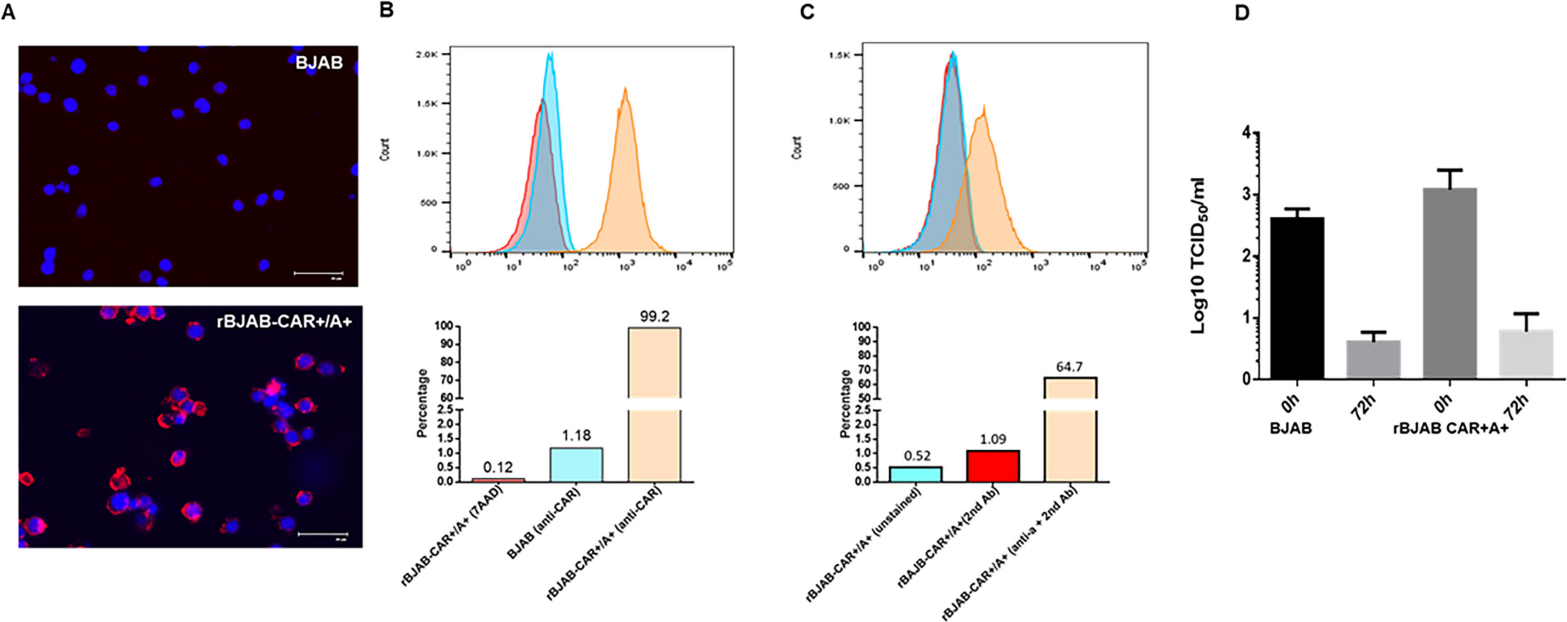
Cell surface expression of CAR and the type A HBGA in rBJAB-CAR+/A + cells. (**A**) Nonpermeabilized BJAB and rBJAB-CAR+/A + cells were stained by the anti-CAR antibody (RmcB) and AF-594 secondary antibody. Cell surface expression of CAR (white arrows) could be detected only in the rBJAB-CAR+/A + cultures. (**B**) Live cell staining with anti-CAR-FITC antibody and flow cytometry analysis revealed cell surface CAR on ~99% of the rBJAB-CAR+/A + cells, while only ~1% of the parental BJAB cells stained positive. (**C**) Live cell staining and flow cytometry analysis of the rBJAB-CAR+/A + cell population with anti-A mouse Mab- and AF488-labeled secondary antibody revealed the presence of cell surface type A HBGA in ~65% of the cells. One of three individual experiments with BJAB-LSU is shown. (D) BJAB and rBJAB CAR +A + cells were infected with ReCV-FT285 (1 MOI), and infectious virus titers were evaluated by titration on LLC-MK2 monolayers at 0 hpi and 72 hpi. The mean and SD of three independent experiments are shown.

Expression vectors carrying the human CXADR cDNA and the alpha-1–3-N-acetylgalactosaminyltransferase cDNA were used to create a recombinant BJAB cell line overexpressing the hCAR and the type A HBGA (rBJAB-CAR+/A+). Immunofluorescence microscopy of unpermeabilized cells did not detect any cell surface CAR in the parental BJAB cell lines, while membrane-associated CAR expression was evident in most of the rBJAB-CAR+/A + cells (Fig. 4A). Flow cytometry analysis of live cells revealed that >90% of the rBJAB-CAR+/A + cells expressed cell surface CAR (Fig. 4B), and 55%–65% of the cell population stained positive for cell surface type A HBGA (Fig. 4C), indicating a significant increase in the cell surface availability of both susceptibility determinants compared to the parental BJAB cell lines. Based on our studies with recombinant CHO cells, cell surface expression of CAR and the type A HBGA is sufficient to convert a nonsusceptible but permissive cell line susceptible to ReCV-FT285 infection (17). However, despite the significant increase in CAR- and type A HBGA-positive cells in the rBJAB-CAR+/A + cell line (Fig. 4A through C), no increase in infectious virus yield was detected compared to the parental cell line (Fig. 4D).

### Preincubation of the ReCV or HuNoV inoculum with *E. cloacae* did not promote infectivity

**Fig 5:**
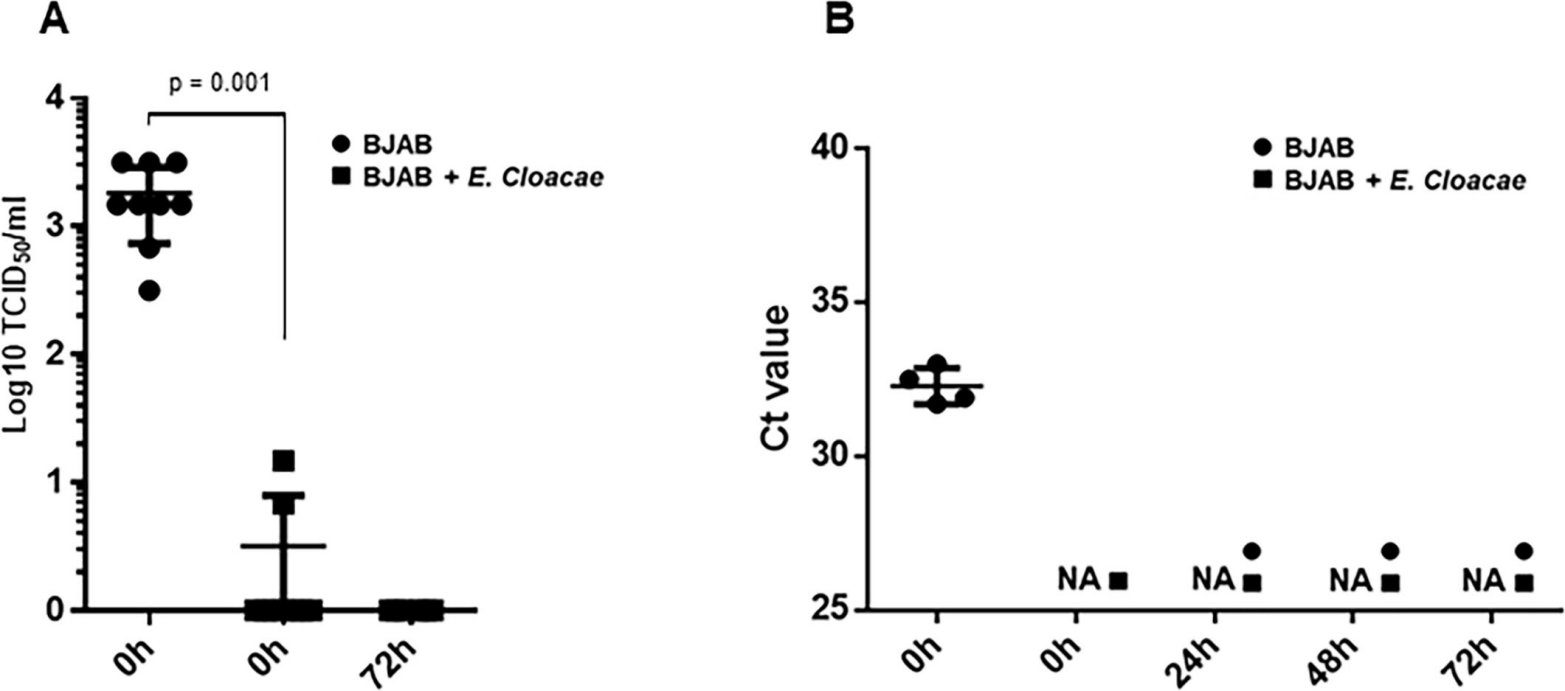
Preincubation of ReCV-FT285 or HuNoV with inactivated *E. cloacae* SENG 6 significantly reduces viral load at 0 hour. (A) BJAB cells were inoculated with 1 MOI of ReCV-FT285 alone or with ReCV-FT285 preincubated with heat-inactivated *E. cloacae* SENG 6 for 1 hour pre-infection. After inoculation and incubation for 1 hour at 37°C, cells were washed three times in culture media by centrifugation for 5 minutes at 700 rpm and plated into 24-well tissue culture plates at a density of 3 × 10^5^ cells/mL. Cultures were harvested immediately after plating (0 hours) and at 72 hours post-infection and titrated on LLC-MK2 cells to determine infectious virus load. (**B**) BJAB cells were inoculated with BCM-HuNoVs with and without preincubation with *E. cloacae* SENG 6, as described in panel A. Samples were harvested immediately after plating (0 hours) and at 24, 48, and 72 hours post-infection and analyzed by qRT-PCR for genome copy number changes. Data shown represent the mean and SD of repeated experiments with the parental and recombinant BJAB-CDC cell clones. Except for the 0 hour time points, there was no difference in the virus yield between the *E. cloacae* SENG 6-treated and untreated samples. NA = no amplification signal was detected. Statistical significance was calculated by two-tailed, unpaired *t*-test using the GraphPad Prism software. A *P*-value ≤ 0.05 was considered statistically significant.

It was reported that preincubation of the HuNoV inoculum with *E. cloacae* or synthetic H-antigen significantly increased infection of BJAB cells (7). ReCV-FT285 and HuNoV were preincubated with heat-inactivated *E. cloacae* SENG 6 and used to infect BJAB cells according to the published protocol (8). This bacterial strain expresses the A, B, and O antigens and binds HuNoVs (18) (Fig. 1F). We did not observe any evidence of infection or virus replication in the cultures. However, a significant difference in virus load in the mock- and bacteria-treated cultures at 0 hour post-infection was evident, indicating that the virus and bacteria (probably as a bacteria–virus complex) were almost completely removed during the wash steps. Thus, while in the cultures without bacteria, both ReCV and HuNoV were able to bind to the cells and remain attached, in the cultures with *E. cloacae* SENG 6, most viral particles attached to the bacteria during preincubation and were washed out during the wash steps. Thus, in this case, the HBGA-expressing bacteria did not help the uptake of the virus and instead of promoting infection prevented attachment to the cells (Fig. 5).

### BJAB cells may not be permissive to ReCV infection

**Fig 6:**
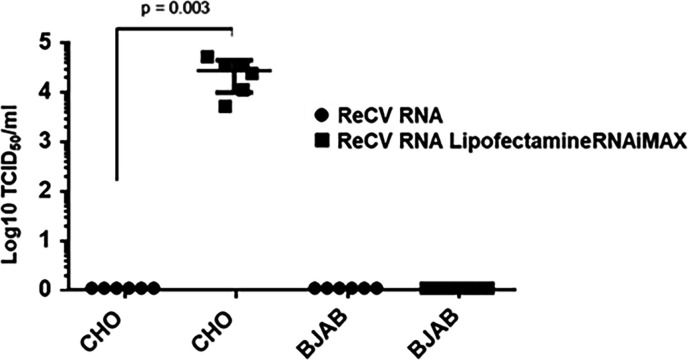
ReCV genomic RNA transfection and infectious virus recovery. BJAB and CHO cells were transfected with ReCV-FT285 genomic RNA and evaluated for the recovery of infectious virus at 72 hours after transfection by titration of cell lysates on LLC-MK2 cells. Cells receiving only viral RNA without the transfection reagent served as the negative control. Data shown represent experiment with BJAB-CDC. Statistical significance was calculated by two-tailed, unpaired *t*-test using the GraphPad Prism software. A *P*-value ≤ 0.05 was considered statistically significant.

BJAB cells were transfected with ReCV-FT285 genomic RNA and monitored for infectious virus recovery to assess their permissiveness to ReCV infection. CHO cells, which are not susceptible but permissive to ReCV infection, were used as the positive control. Positive-sense RNA virus genomes containing a 5’-cap or in the case of caliciviruses Vpg (viral protein genome linked) and a 3’-poly-A tail are functional mRNAs. Since the Vpg is covalently linked to the genome, the calicivirus genomic RNA extracted by conventional RNA extraction methods remains a fully functional mRNA. After 72 hours post-transfection of BJAB and CHO cells with viral genomic RNA, cultures were evaluated for the presence of infectious virus by titration in LLC-MK2 cells. Infectious virus could be recovered only from the CHO cell cultures that were transfected with ReCV genomic RNA and lipofectamine RNAiMax but not from CHO cells that received only ReCV RNA without the transfection reagent or from any of the BJAB cultures, indicating that BJAB cells are not permissive to ReCV infection (Fig. 6). We could not evaluate HuNoV permissiveness due to the lack of a sensitive cell culture assay to detect infectious virus recovery.

**Fig 7:**
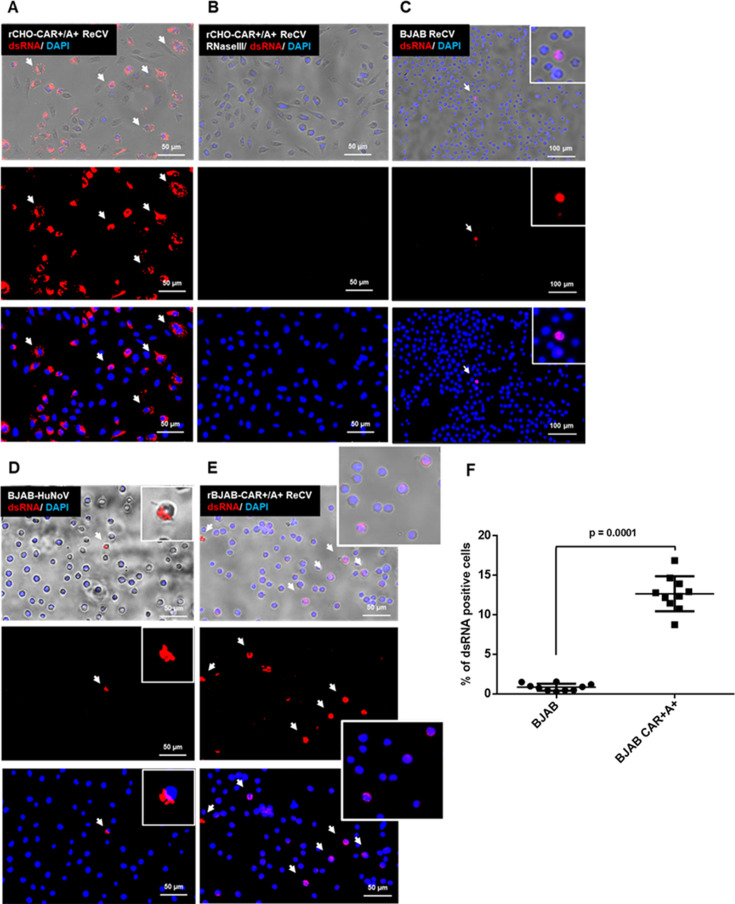
Detection of replication intermediate dsRNA in ReCV- and HuNoV-infected cells. rCHO-CAR+/A + and BJAB cell cultures were infected with ReCV-FT285 (1 MOI) or GII.4 [P31]/BCM16-2 HuNoV (10^5^ genome copy/well) and stained with dsRNA-specific J2 antibody at 6 hours post-infection. rCHO-CAR+/A + cells infected with ReCV-FT285 served as the positive control. The specificity of dsRNA staining was evaluated by treating the infected cells with RNase III before J2 antibody staining (only rCHO-CAR+/A + is shown) (panels A and B). A small number of ReCV- or HuNoV-infected parental BJAB cells (≤ 1 cell per view field) stained positive for dsRNA (panels C and D). The number of dsRNA-positive cells in the ReCV-FT285-infected rBJAB-CAR+/A + cultures was significantly higher (panels E and F). White arrows indicate dsRNA-positive cells. HuNoV BJAB cell infections were performed according to the protocol described by Jones et al. (8). A representative of three repeated experiments with BJAB-CDC is shown. Panel F was created based on 10 random images taken at the same magnification of three repeated infection/IFA staining experiments. Total and dsRNA-positive cell counts were determined by using the ImageJ software. Statistical significance was calculated by two-tailed, unpaired *t*-test using the GraphPad Prism software. A *P*-value ≤ 0.05 was considered statistically significant.

Anti-dsRNA antibody was used to evaluate the presence of intermediate viral dsRNA in infected cultures at 6 and 9 hours post-infection. A rCHO cell line was used as a positive control (17, 19). The J2 antibody specificity was demonstrated by RNase III treatment of infected cultures before staining. Multiple dsRNA-positive cells were observed per view field in the ReCV-infected rCHO cell cultures, and RNase III treatment significantly reduced or eliminated the signal, confirming the specificity of the J2 antibody to dsRNA (Fig. 7A and B). In contrast, in both the ReCV- or HuNoV-infected BJAB cultures, only a few dsRNA-positive cells were observed per view field (Fig. 7C and D). Interestingly, when rBJAB-CAR+/A + cells were evaluated, the number of dsRNA-positive cells increased significantly (8–10 per view field) (Fig. 7E), but without the increase in the virus yield at 72 hours (Fig. 4D).

## DISCUSSION

Since the discovery of the prototype Norwalk virus in 1972 (20), the lack of a cell culture system represented a major bottleneck for the progress in HuNoV research. The discovery of murine norovirus (MNV) as the first cell culture propagable norovirus with a tropism for dendritic cells and macrophages (3) suggested the possibility of immune cell tropism of HuNoVs. Animal challenge studies and analysis of biopsy samples of HuNoV-infected patients demonstrated the presence of HuNoV antigens in immune cells of the intestinal lamina propria, including dendritic cells, macrophages, and B cells (4–6). All of these cells are antigen-presenting cells (APC), which could take up and process viral antigens, without being infected. *In vitro* studies using PBMCs derived from susceptible individuals failed to demonstrate HuNoV replication in human dendritic cells and macrophages (21). HuNoV immune cell tropism remained elusive until 2014, when Jones et al*.* reported that human B cell lines, including BJAB, could support the replication of a human GII.4 norovirus and announced the development of the first HuNoV cell culture system (7). However, later several technical issues were noted, including low and inconsistent virus yield and poor reproducibility among different laboratories (8), which led to a debate about the validity and applicability of the B cell culture for HuNoV research and the immune cell tropism of HuNoVs. While the two original B cell papers (7, 8) collected over 1,000 citations, to our knowledge, very few other studies used this system in HuNoV research (22), even in the laboratories that described the system. Meanwhile, the enterocyte tropism of HuNoVs and the enteroid culture system gained large-scale acceptance and use with numerous manuscripts published worldwide (10, 14, 23), even though this system neither represents a robust nor efficient cell culture for HuNoV.

We must note that a recent study investigating HuNoV replication in human B cells derived either from whole-blood PBMCs or from spleen and lymph nodes demonstrated a 3–18-fold HuNoV genome copy number increase, and treatment with the nucleoside analog 2′CMC or type I IFN abolished this, suggesting that B cells are susceptible and at least partially permissive to HuNoV infection. However, the low levels of viral genome copy number and dsRNA increases suggested that HuNoV infection in B cells is likely defective, resulting in an abortive infection (24). This observation seems to further challenge the feasibility of B cell cultures as an efficient HuNoV cell culture system. However, we also have to consider that transformed cells often accrue significant gene expression profile changes that could lead to changes in the susceptibility to certain infections that normal cells are resistant to.

Currently, our understanding of the susceptibility determinants of HuNoV infections is limited. While there is strong evidence for the role of HBGAs and other cell surface carbohydrate molecules (e.g., sialic acid) in strain-specific susceptibility, it is evident that HBGAs are not the entry receptors of HuNoVs, and cell surface expression of HBGAs is not enough to promote susceptibility to infection (25).

In our previous studies, we described remarkable biological similarities between HuNoVs and ReCVs, including strain-specific diverse HBGA binding (26, 27). In a rhesus macaque challenge study, we reported the presence of Tulane virus antigen in CD20 +positive B cells in the lamina propria of duodenal biopsies. Similarly, Tulane virus antigen was detected in CD20 +but not in CD3 +cells in rhesus PBMC cultures, and a slight increase in Tulane virus genome copy numbers was also noted (15). In addition, recently, we identified the ReCV entry receptor, (16) and using a panel of recombinant CHO cell lines, we characterized the minimum requirements for susceptibility for three GI ReCV strains with diverse *in vitro* HBGA binding (17). We found that the cell surface expression of CAR and either type A or B HBGAs or sialic acid controls strain-specific susceptibility to ReCV infection. This presented the opportunity to re-evaluate ReCV B cell infections in correlation with the cell surface expression of susceptibility determinants that could also provide valuable clues for improving HuNoV B cell cultures.

We selected the ReCV-FT285 strain that relies on CAR and the type A or B HBGAs for infection, but not sialic acids that are abundant in most cell lines (17). Three NHP origin B cell lines and the human BJAB cell line were tested in infection experiments. Since BJAB cell lines are frequently shared among laboratories with no clear records of their original source, we evaluated two BJAB clones from different sources, one of which was used for establishing the HuNoV B cell culture (7). A 24-loci short tandem repeat (STR) analysis to assess cell line identity was performed and yielded a 98.36% CLASTR result score to the BJAB (CVCL-5711) reference cell line for both BJAB clones used in our study.

We found no evidence of ReCV-FT285 replication in any of the NHP B cell lines, as indicated by the gradual decrease in virus titers post-infection (Fig. 1B). Neither CAR nor HBGA expression was detected in any of the NHP B cell lines (Fig. 1E and F). Although it was beyond the scope of this study to investigate, it is possible that some of the NHP B cell lines could become susceptible to ReCV infection if they expressed CAR or A/B HBGA.

Contrarily, virus titers in the infected BJAB cell cultures exhibited an initial eclipse phase at 12 hours post-infection, followed by a modest uptick that stabilized at about the initial 0 hour titer by 48–72 hours post-infection (Fig. 1C). Based on qRT-PCR, there was a slight genome copy number increase at 72 hours post-infection (3–4 Ct value difference) that could not have been picked up in the TCID_50_ assay with 10-fold dilution series (Fig. 1D). This pattern agreed with an initial phase of replication that subsequently became abortive. Evaluation of CAR and HBGA expression by Western or slot blotting revealed CAR and HBGA expressions in the BJAB cell lysates (Fig. 1E and F). However, the relatively high expression level of CAR and HBGAs in the BJAB cells was in contrast with the notably low virus yield, which could have been explained by i) BJAB cells are nonpermissive to ReCV infection or ii) CAR and/or HBGAs are not accessible to the virus. Indeed, live cell staining and flow cytometry analysis revealed that less than 1% of the BJAB cells had cell surface CAR or HBGAs (Fig. 2A and B), while over 80% of permeabilized cells stained positive for CAR (Fig. 2C). Thus, while CAR is expressed in the BJAB cell clones, it remains internalized and inaccessible for virus entry.

Both CAR and HBGA expressions in normal hematopoietic cells remain poorly understood. CAR mRNA has been identified in CD34 +hematopoietic stem cells (28), and analysis of freshly isolated bone marrow cells revealed CAR expression on differentiated erythroid and myeloid cells, a subset of CD34 +progenitor cells, but not on CD19+, CD4+, and CD8 +lymphoid cells (29). In contrast, CAR expression has been reported in various transformed B cell lines (30). In a study of adenovirus latency within human lymphoid cell lines (Ramos, BJAB, and KE37), 60%–90% of the cells initially expressed cell surface CAR (31). This is markedly higher than the less than 1% cell surface CAR +BJAB cells observed in our study (Fig. 2A). Notably, all lymphoid cell lines experienced a loss of CAR expression within 24 hours of adenovirus infection, and the number of CAR-expressing cells remained low in the persistently infected cell populations for up to a year. Moreover, these cells were resistant to reinfection by adenovirus, even after reintroducing cell surface CAR via retroviral transduction, indicating stable alterations in the expression of multiple genes, not just CXADR (31). We speculate that the two BJAB clones used in our study may harbor similar genetic or epigenetic modifications, potentially due to persistent viral infections, which could be investigated in future studies.

The expression of ABH antigens on normal peripheral lymphocytes is highly debated. Lymphocytes from type A or B secretor individuals (FUT2+) test positive for ABH antigens, whereas those from ABH non-secretors (FUT1 +FUT2-) do not. Several studies have demonstrated the *in vitro* transfer of ABH antigens from serum to lymphocytes, suggesting that lymphocytes do not synthesize ABH and Lewis antigens but rather adsorb them from the bloodstream (32–35). We attempted to enrich cell surface HBGAs on BJAB cells by incubation in human AB serum, but our efforts were unsuccessful (Fig. 3B).

We were able to significantly enhance the cell surface expression of CAR and the type A HBGA using expression vectors in both parental BJAB clones (Fig. 4A through C). However, despite the significant increase in the expression of cell surface entry receptors, infection of the rBJAB-CAR+/A + cells did not result in a higher infectious virus yield compared to the parental cells (Fig. 4D).

Based on current knowledge regarding the expression of CAR and HBGA in lymphocytes, B and T cells are unlikely to be targeted by ReCV infections. Nonetheless, there is a possibility that a specific group of lymphocytes residing in the intestinal tissue may express these ligands. Additionally, it has been shown that viral transformation of lymphocytes, such as with EBV, can lead to atypical expression of these antigens (36).

Since it was reported that enteric bacteria expressing the H antigen promote GII.4 HuNoV infection in B cells (7), we evaluated whether *E. cloacae* SENG 6, a HBGA-expressing strain, promotes ReCV or HuNoV infection. We used heat-inactivated *E. cloacae* SENG 6 according to the original protocol described by Jones et al*.* (8). We did not observe any evidence suggesting that bacteria promote infectivity. On the contrary, we observed significantly lower virus loads in *E. cloacae-*treated cultures compared with cultures without bacteria at 0 hours, indicating that the bacteria bound both viruses during the preincubation period, prevented virus binding to cells, and bacteria–virus complexes were removed during the wash steps (Fig. 5). Thus, in our case, the HBGA-expressing bacteria did not help but prevented both ReCV and HuNoV attachment and uptake by BJAB cells. Similarly, in a study using a gnotobiotic (Gn) pig model, *E. cloacae* inhibited HuNoV infection *in vivo*, and viral infection of B cells was not observed with or without the presence of *E. cloacae* (37). We must note that the *E. cloacae* strain used in our study differed from the strain used in the original study.

Since CAR and type A or B HBGA expression fully supported ReCV-FT285 infection in CHO cells, we suspected that BJAB cells are not permissive to ReCV infection. To evaluate this, we transfected BJAB cells and CHO cells with ReCV genomic RNA and analyzed the recovery of infectious virus in LLC-MK2 cells as previously described (16). While CHO cells produced infectious virus, BJAB cells did not (Fig. 6), indicating the lack of permissiveness of BJAB cells. However, we also must consider the possibility that only a small subset of the BJAB cells is permissive, and this coupled with a lower than 100% transfection efficiency did not produce a detectable amount of infectious virus.

To further evaluate the degree of susceptibility to ReCV and HuNoV infections, we evaluated the presence of viral replication intermediate dsRNA at 6 hours post-infection in BJAB cell cultures. The presence of dsRNA in infected cells indicates successful entry, uncoating, polyprotein processing, and genomic RNA replication in the calicivirus replication cycle. In both ReCV- and HuNoV-infected parental BJAB cultures, only a few cells stained positive for dsRNA (0.91 ± 0.48 cells/view field), while in the ReCV-infected rBJAB-CAR+/A + cultures, the number of dsRNA positive cells significantly increased (12.81 ± 2.46 cells/view field) (Fig. 7). However, this did not lead to an increased infectious ReCV yield compared to the parental cell line or over the input virus at later time points post-infection (Fig. 4D).

These observations further support an initial infection of the limited number of susceptible BJAB cells that is interrupted and transitions to an abortive infection cycle.

In summary, our investigations have not revealed any significant ReCV or HuNoV infection or replication in the B cell lines examined. Although low-level initial infection was observed in BJAB cell lines, the infection did not progress. For ReCVs in BJAB cells, this could be attributed to the impaired cell surface expression of CAR and HBGAs. The specific susceptibility determinants for HuNoV infection have yet to be determined. While the B cell tropism of HuNoVs and ReCVs may remain a subject of debate, the concept that B cell lines represent an efficient HuNoV or ReCV cell culture system is not warranted, and until major improvements in reproducibility and virus yield are achieved, they should not be referred to as such.

## MATERIALS AND METHODS

### Virus strains

Experiments were performed with ReCV-FT285 (KC662366, GI.2). This strain requires cell surface expression CAR and the type A or B HBGA for attachment and entry, and it does not utilize sialic acid (17). Virus stocks were grown in LLC-MK2 cells, filtered through 0.2 µm vacuum filter units, titrated (TCID_50_/mL), aliquoted, and stored at −80°C until used. A purified HuNoV-positive sample [GII.4 [P31]/BCM16-2] with a 6.30E + 05 /µL genome copy titer was obtained from Dr. Mary Estes and stored in 5 µL aliquots at −80°C.

### Cell lines

LLC-MK2 cells were maintained in M199 medium supplemented with 10% FBS and penicillin/streptomycin/amphotericin B (P/S/A). Nonhuman primate B cell lines, including BLCL-C162 (rhesus macaque), V038 AGM BLCL (African green monkey), and AG23 AGM BLCL (African green monkey), were obtained from the NHP Reagent Resource. BJAB cells were obtained from Dr. Rhonda Cardin (designated in our study as BJAB-LSU) and Dr. Jan Vinje (designated as BJAB-CDC). The latter was the same cell line that was used in the original work describing the human norovirus BJAB culture (7). Recombinant BJAB cell lines overexpressing hCAR and the type A HBGA were created in this study by electroporation (Gene Pulser Xcell Electroporation System, Bio-Rad) of expression vectors pcDNA3.1/zeo(+) carrying the human CXADR cDNA and pcDNA3.1/neo(+) carrying the human alpha-1–3-N-acetylgalactosaminyltransferase cDNA into BJAB-LSU and BJAB-CDC cells. Transfected cells were selected by the corresponding selection drugs, and cell surface expressions of CAR and type A HBGA were evaluated by immunofluorescence staining of unpermeabilized cells and by flow cytometry analysis and sorting of live cells. All B cell lines were cultured in RPMI-1640 medium with L-glutamine, supplemented with 10% FBS and P/S/A, as described by Jones et al. (8).

### Cell line authentication

Since the original sources and history of the BJAB cell lines could not be established, a 24 loci short tandem repeat (STR) analysis was performed to assess cell line identity. Data were analyzed using the CLASTR 1.4.4-STR similarity search tool (https://www.cellosaurus.org/str-search/). Both the cell lines yielded a 98.36% CLASTR result score to the BJAB (CVCL-5711) reference cell line in the database.

### Cell viability

Cell viability in all treated and mock cultures was evaluated by the trypan blue exclusion assay. Only B cell cultures with round cells displaying sharp, shiny periphery, forming small clumps, and containing <3% dead cells were used for infection studies (Fig. 1A).

### Antibodies and reagents

Anti-CAR monoclonal antibody RmcB (Millipore Sigma, Cat# 05–644) and F(ab')2-Goat anti-Mouse IgG (H + L) Cross-Adsorbed Secondary Antibody, Alexa Fluor 594 (Invitrogen, Cat# A-11020) were used for the immunofluorescence detection of CAR. Anti-A (ABO1) (Diagast, Cat# 70501) and anti-B (ABO2) (Diagast, Cat# 70502) mouse monoclonal antibodies (IgM) and goat anti-Mouse IgM (Heavy chain) Cross-Adsorbed Secondary Antibody, Alexa Fluor 568 (Invitrogen, Cat# A-21043), were used for the immunofluorescence detection of HBGAs. Anti-dsRNA monoclonal antibody (clone J2, Scicons) was used to detect viral dsRNA intermediates. RNase III (ThermoFisher Scientific, Cat# AM2290) was used to test J2 antibody specificity. In Western blots, CAR was detected by a rabbit anti-CAR polyclonal antibody (ThermoFisher Scientific, Cat# PA5-31175) and goat anti-Rabbit IgG (H + L) Cross-Adsorbed Secondary Antibody, HRP (Invitrogen, Cat# G-21234).

Flow cytometry reagents including flow cytometry staining buffer (Cat# 420201), human true stain FcX Fc receptor blocking solution (Cat# 422301), and 7-AAD viability staining solution (Cat# 420403) were purchased from BioLegend. Recombinant anti-CXADR/CAR antibody (FITC) (SinoBiological, Cat# 10799-R271-F) and anti-A (ABO1) and anti-B (ABO2) (Diagast, Cat# 70501 and 70502) mouse monoclonal antibodies (IgM) were used for CAR and type A or type B HBGA staining, respectively. For HBGA detection, Alexa Flour 488 anti-mouse IgM antibody (BioLegend, Cat# 406522) was used as the secondary antibody.

### Infection of B cells

Culturing of B cell lines and infection procedure followed the protocol described by Jones et al., with the exception that cells were washed three times with 1 mL culture media after incubation with the virus or virus/inactivated bacteria mixture (8). MOIs are listed in figure legends based on TCID_50_ for ReCV or genome copy number for HuNoV. For bacterial stimulation of virus infection, heat-inactivated *Enterobacter cloacae* SENG-6 was used. The extracellular polymeric substance (EPS) of this bacterium contains high levels of HBGAs (18). Inactivated bacteria were tested for the expression of type A and B HBGA in slot blots (Fig. 1F).

### Virus titration

LLC-MK2 cells (1 × 10^4^ cells/well) seeded in 96-well plates were inoculated with tenfold serial dilutions of samples (100 µL/well, three wells/dilution). Plates were incubated for 5 days and stained with crystal violet. Virus titers (TCID_50_) were calculated using the Reed and Muench method.

### qRT-PCR

The iTaq Universal SYBR Green One-Step Kit (Bio-Rad, Cat# 1725150) and an ReCV-FT285 specific primer pair (FT285F: 5’- CCTAAGGCACCTGAGCTTATTG −3’ FT285R: 5’- GAGAAGTGAAGTGGTTGGGATG −3’) were used for ReCV detection. The Reliance One-Step Multiplex Supermix (BioRad, Cat# 12010176) and GII human norovirus primer set and probe (COG2F, COG2R, and RING2) adopted from Kageyama et al*.* (38) were used for HuNoV detection. Reactions were run on a CFX Opus 96 real-time PCR system (BioRad) using the preprogrammed protocol for each kit. Results were analyzed using the CFX Maestro 2.3 software (BioRad). An increase or decrease in the genome copy number was determined based on a decrease or increase in Ct values between the 0 hour and corresponding samples at different time points post-infection.

### Immunofluorescence

B cells were washed in PBS and deposited onto chamber slides. After 30 minutes of incubation, the PBS was gently removed, and the sedimented cells were fixed with 4% paraformaldehyde (PFA) with or without permeabilization with 0.1% Triton X-100 (cell surface vs intracellular staining). For dsRNA detection, control wells were treated with RNase III (10 U/mL, 150 µL/well) for 2 hours at 37°C. Slides were blocked with PBS-3%; BSA-10% human serum (for FcR blocking) for 2 hours at room temperature. Antibodies were diluted in PBS-3%; BSA-0.1% Tween 20. Slides with primary antibodies were incubated in a humidifier box overnight at 4°C. Washes were done with PBS-0.1% Tween 20 (PBS-T, pH 7.4). Slides overlayed with secondary antibodies were incubated for 1.5 hours at 37°C, washed, mounted with coverslips using ProLong Diamond Antifade Mountant with DAPI (ThermoFisher Scientific, Cat# P36962), and cured overnight in the dark at room temperature. Images were captured on an Evos FLoid Cell Imaging Station (Life Technologies).

### Western/slot blot analysis

Cells were washed with ice-cold PBS, counted, and 2 × 10^6^ cells were lysed in 0.3 mL Pierce RIPA buffer (ThermoFisher Scientific, Cat# 89900) containing the Halt protease inhibitor cocktail (ThermoFisher Scientific, Cat# 87786). The lysates were incubated on ice for 30 minutes with vortexing every 5 minutes. The insoluble material was removed by centrifugation at 12,000 rpm for 15 minutes at 37°C, and samples were aliquoted and stored at −80°C until used. The protein concentration was determined by the Bradford protein assay (Bio-Rad, Cat# 5000002). For analysis, samples (~20 µg protein) were mixed with an equal volume of 2X Laemmli sample buffer (Bio-Rad, Cat# 1610737), boiled for 5 min, separated on 4%–20% Mini-Protean TGX precast protein gels (Bio-Rad, Cat#: 4561093), and transferred to polyvinylidene difluoride (PVDF) membranes (Bio-Rad, Cat# 1620174). For slot blots, a vacuum manifold was used. Membranes were blocked with 5% nonfat dried milk in PBS-T, at room temperature, for 3 hours, rinsed with PBS-T, overlayed with the primary antibody (0.25 µg/mL), diluted in blocking buffer, incubated with agitation at 4°C overnight, and washed with PBS-T. Membranes were incubated with the HRP-conjugated secondary antibody for 3 hours at room temperature and washed with PBS-T. Signals were detected with SuperSignal West Pico PLUS chemiluminescent substrate (ThermoFisher Scientific, Cat# 34577) using a ChemiDoc MP imaging system (BioRad). Subsequently, Western blot membranes were stripped using Restore PLUS Western Blot stripping buffer (ThermoFisher Scientific, Cat# 46430) and re-probed with mouse monoclonal antibody to GAPDH (ThermoFisher Scientific, Cat# MA5-15738).

### Flow cytometry

B cells (4 × 10^6^ cells) were collected on ice in 1.5 mL microcentrifuge tubes, pelleted at 400 *g* for 5 minutes at 4°C, and washed with 1 mL flow cytometry staining buffer (FACS buffer). For the analysis of fixed, permeabilized cells, the cell pellet was fixed in 200 µL of 4% PFA for 20 minutes at RT, washed twice with FACS buffer, and permeabilized in 200 µL of PBS-0.1% Triton X-100 for 15 minutes at RT. Both live and fixed B cells were resuspended in 50 µL FACS buffer containing 0.5 µL human true stain FcX Fc receptor blocking solution. After incubation for 10 minutes at RT, 50 µL of the primary antibody diluted in FACS buffer was added to the samples, and tubes were incubated in the dark for 1 hour at RT. After washing the cells in FACS buffer, the cells stained for CAR detection (FITC-labeled primary antibody) were resuspended in 0.5 mL FACS buffer containing 5 µL 7-AAD viability staining solution and analyzed. Samples stained for HBGA detection were resuspended in 50 µL FACS buffer containing 0.5 µL human true stain FcX Fc receptor blocking solution, followed by the addition of 50 µL of diluted secondary antibodies, incubated in the dark for 45 minutes at RT, washed with FACS buffer, resuspended in 0.5 mL FACS buffer containing 5 µL 7-AAD, and analyzed. Samples were run on a Beckman Coulter MoFlo Astrios cell sorter using the Summit version 6.3 acquisition program (Beckman Coulter Inc., Brea, CA). Data were analyzed using FlowJo version 10.8.1 (Becton Dickinson Biosciences, San Jose, CA).

### Transfection of BJAB cells with ReCV genomic RNA

ReCV-FT285 RNA was extracted from virus stocks using the TRIzol LS reagent (Thermo Fisher Scientific, Cat# 10206010) according to the manufacturer’s protocol. Then, 1.5 × 10^5^ BJAB cells in 24-well tissue culture plates were transfected with 100 ng total RNA using the Lipofectamine RNAiMAX transfection reagent (Thermo Fisher Scientific, cat: 13778150) according to the manufacturer’s instructions. As a positive control, CHO cell monolayers containing the same number of cells were used. Cells receiving 100 ng total RNA without the transfection reagent served as a negative control. Plates were incubated for 72 hours post-transfection, subjected to three freeze–thaw cycles, and harvested into 1.5 mL Eppendorf tubes. Cell debris were removed by centrifugation, and supernatants were titrated on LLC-MK2 cells in 96-well plates to assess the recovery of infectious virus.

### Statistical analyses

Experiments were performed in at least three independent replicates. Differences between virus titers were evaluated for statistical significance by unpaired *t*-test using the GraphPad Prism software (Dotmatics). A *P*-value ≤ 0.05 was considered statistically significant.

## Data Availability

The data supporting the findings of this study have not been deposited in public repositories but are available from the corresponding author upon reasonable request.
